# Patrimony and the Evolution of Risk-Taking

**DOI:** 10.1371/journal.pone.0011656

**Published:** 2010-07-19

**Authors:** Michael D. Stern

**Affiliations:** Laboratory of Cardiovascular Science, National Institute on Aging, National Institutes of Health, Baltimore, Maryland, United States of America; CNRS, France

## Abstract

The propensity to make risky choices has a genetic component, and recent studies have identified several specific genes that contribute to this trait. Since risk-taking often appears irrational or maladaptive, the question arises how (or if) natural selection favors risk-taking. Here we show, using a stochastic simulation of selection between two hypothetical species, “R” (risk-seeking) and “A” (risk-averse) that, when expected reproductive fitness of the individual is unaffected by the making of the risky choice (winnings balanced by losses) natural selection (taken to the point of extinction) favors the risk-averse species. However, the situation is entirely reversed if offspring are permitted to inherit a small fraction of the parent's increased or decreased fitness acquired through risk-taking. This seemingly Lamarckian form of inheritance actually corresponds to the human situation when property or culture are transmitted in families. In the presence of this “cultural inheritance”, the long-shot risk-taking species was overwhelmingly favored, even when 90% of individuals were rendered sterile by a losing choice. Given this strong effect in a minimal model, it is important to consider the co-evolution of genes and culture when interpreting the genetics of risk-taking. This conclusion applies, in principle, to any species where parental resources can directly affect the fecundity of offspring. It might also be relevant to the effects of epigenetic inheritance, if the epigenetic state of zygotes can be affected by parental experiences.

## Introduction

The recent financial crisis has shone a bright light on the tendency of humans to take apparently irrational risks. The propensity to choose risky behaviors in social situations (impulsiveness, addiction, conduct disorder) and in economic decisions (gambling) is known to be influenced by genetic factors [1]. Twin studies have shown that 20% of the variation in financial risk-taking in experimental lotteries is genetic [2], as is 35–54% in compulsive gambling [3]. Recently, particular alleles of several genes involved in processing of neurotransmitters – monoamine oxidase (MAOA), the serotonin transporter (5-HTTLPR) and a dopamine receptor (DRD4) – have been implicated in risk-taking in behavioral disorders and in experimental financial investing models, and in gambling [4]–[7]. It is likely that many other genetic influences remain to be discovered.

While it has been difficult to establish unambiguous animal models of gambling behavior [8]–[11], a natural analog exists, in the form of risk-sensitive foraging [12]. If individual animals are confronted with a choice between two feeding alternatives, one of which provides a certain amount of food, while the other offers an uncertain yield with the same expected value, they will commonly show a preference for high or low variance. These traits of risk-seeking or risk aversion are widespread across phyla, and have been shown to be partly genetic, though also influenced by environment and the history of the individual animal [12].

The genetic component of risk-sensitivity might be a coincidental side-effect of selection of genes for other functions in the nervous system – a *spandrel* in Stephen Jay Gould's terminology. However, in view of the critical importance of risk-management for the survival of organisms, it is reasonable to suspect that it is adaptive, i.e. produced by natural selection. Theories of adaptive risk-sensitive foraging [12]–[15] have assumed that the animal implicitly optimizes a currency (e.g. average rate of energy intake) which is a surrogate for reproductive fitness, the ultimate currency of evolution. The latter is usually taken to mean expected number of offspring surviving to reproduce. If the expected yield of foraging were itself being optimized, then its variance should have no effect. If, however, the relationship between foraging yield and reproductive fitness is non-linear – for example if the animal will starve to death if the current feeding opportunity yields no more than its expected value – then risk-seeking or risk-averse behavior would be selected, depending on the direction of the non-linearity. A similar argument has been applied in the classical economic theory of maximization of expected personal utility [16], in which risk aversion is attributed to decreasing marginal utility as a function of wealth, while long-shot gambling is postulated to be due to a hypothetical convexity of the utility function at very high returns.

Such analyses assume that natural selection is tantamount to rational optimization of the expected number of offspring of an individual organism. To seek the possible origin of risk-seeking when there is no apparent benefit, we asked whether the stochastic process of evolution itself, operating over many generations, would show a preference for high or low risk in a “fair” match-up in which the expected number of offspring of each organism was unaltered by taking the risk. To examine this question in a simple model, we performed a stochastic, numerical simulation of the competition between two species which differed in their propensity to accept a “fair gamble” in which the single-generation fitness gain of a “win” was balanced, in expectation, by the cost of a loss. We found a clear effect of risk *per se*, but the direction of the effect was strongly influenced by the inclusion of non-genetic inheritance in the model.

## Results

We constructed a computational model of selection in which two non-sexual “species” “R”(risky) and “A”(averse) compete on equal terms. Each organism begins with the same intrinsic fecundity *g*_0_, (the term fecundity is used here to include both survival and reproduction over a single generational cycle) but each “R” organism in each generation experiences a “risk event” in which it may either have its fecundity boosted by a reward factor *r*>1, with probability *p*, or reduced by a penalty factor (1−*rp*)/(1−*p*) with probability (1−*p*). It is easily verified that the expected value of *g* is unchanged by this choice, provided that *r*<1/*p* to avoid the possibility of negative fecundity. The maximum risk *r* = 1/*p* corresponds to the situation in which a loser has no chance to reproduce. Symmetrical competition for resources was enforced by dividing all fecundities by (1+*n*/*n_max_*) where n is the total population of the current generation (both “R” and “A”) and *n_max_* is a population scale. For the case *g*_0_ = 2, *n_max_* approximates the “carrying capacity” of the “environment”, and would be the saturation population size if the model were deterministic (logistic growth). The actual, realized number of offspring that each organism contributes to the next generation is then chosen from a Poisson distribution with mean equal to that organism's scaled fecundity *w* (the absolute fitness according to traditional definitions).

**Figure 1:**
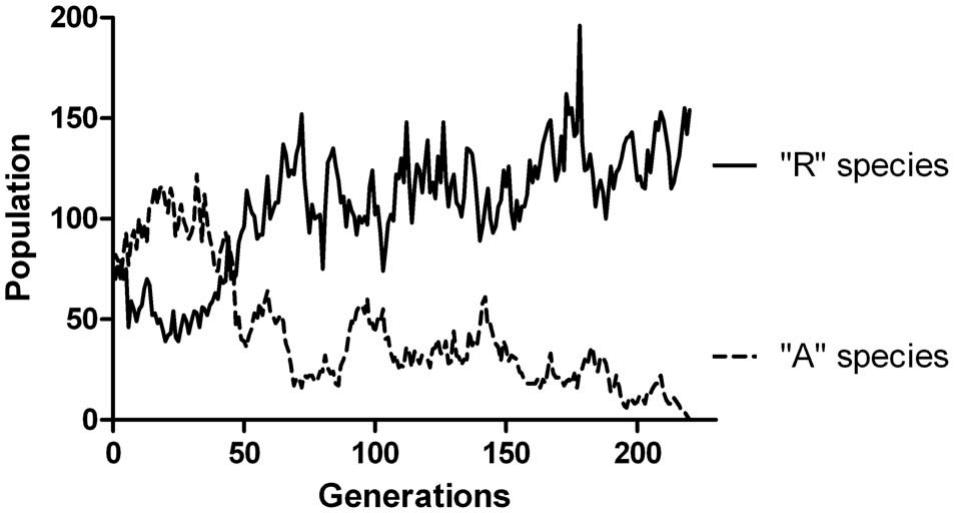
Trajectory of the populations of two species in the stochastic selection model. The species “R” (solid line) and “A” (dashed line) were actually identical because the risk-reward *r* was set to 1. Both species had a baseline fecundity *g*_0_ = 2, *i.e.* in the absence of risk and competition, each organism would produce a mean of 2 offspring. The population ceiling parameter *n_max_* was set to 150. The model was run until one species (“A”) became extinct, after 220 generations.

When *r* = 1 the two species are, in fact, identical since the risk event then has no effect on fecundity. Competition between identical species for a single ecological niche is unstable due to stochastic drift, and one or the other species will eventually go extinct. For a modest population size (150) this generally took less than 200 generations. A typical population trajectory is shown in Figure 1. The model was run until one species went extinct, and this run was repeated 10,000 times for each chosen combination of *r* and *p*, in order to estimate the frequency with which “R” (risk-seeking) organisms are the winners of “natural selection.” There is no mutation or evolution per se in this model; we model only the process of competitive selection.

**Figure 2:**
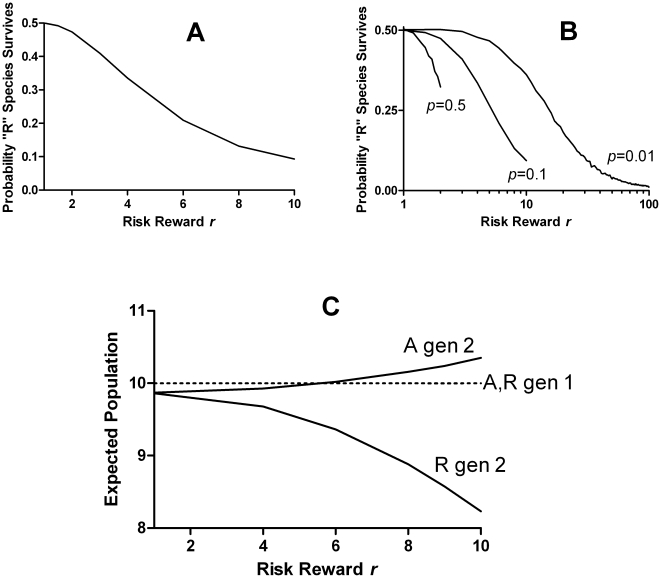
Intrinsic selection against risk-taking species. A. The fraction of 10,000 trials in which the “R” species survived the selection, as a function of the risk-reward *r*. The probability *p* of the risk event was kept constant at 0.1, and *n_max_* at 150 Higher values of *r* indicate a higher reproductive advantage for the 10% of “R” organisms that “win” the risk event, but also a correspondingly greater penalty for the 90% that lose, so that the overall expected fecundity remains at 2. When *r* = 1/*p*, the losers leave no descendants. B. Similar to A, but showing different values of the long-shot probability *p*. C. Analytically computed expectation values of the populations of “R” and “A” organisms after 1 or 2 generations, starting from 10 of each, with the deterministic saturation population size set to 20 and *p* = 0.1. The effect of risk to depress the expected “R” population appears only in the second generation, due to correlation between fecundity fluctuations in the first generation and population competition in the second. Note that even when *r* = 1 both populations are slightly depressed due to the effect of Poisson fluctuations in individual fecundity.

**Figure 3:**
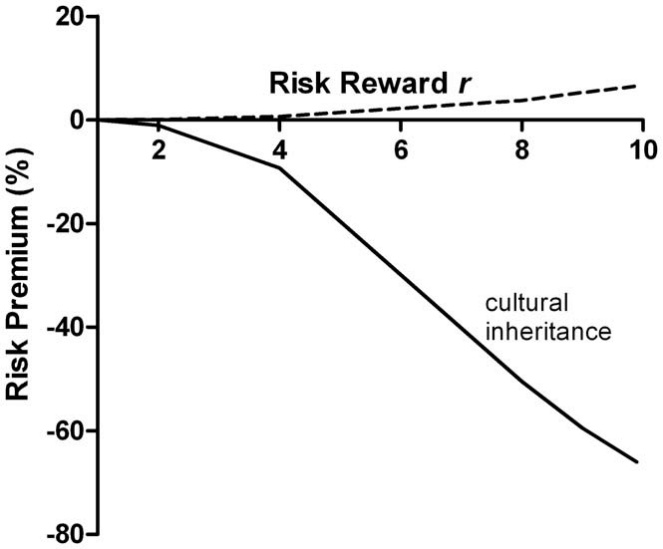
Risk premium/discount. *Dashed curve*: The selective disadvantage of the risk-taking “R” species, expressed as a “risk premium”: the percentage increase in baseline fecundity of “R” needed to maintain a 50-50 split in the outcomes of selection trials. *P* = 0.1, *n_max_* = 150 *Solid curve*: In the presence of “cultural inheritance” (α = 0.091 corresponding to a wealth persistence time of 1.1 generations) the risk “premium” is strongly negative, *i.e.* a discount, indicating that the propensity to take even an unfair gamble is positively selected.

As shown in Figures 2A and B, while the success rate of “R” was 50% when *r* = 1, as required by symmetry, it declined at higher risk levels, even though the single-generation expected fecundity was unchanged. In other words, stochastic evolutionary selection is itself risk-averse. As shown in Figure 2B, the magnitude of this risk aversion depended on the relationship between the risk reward factor *r* and the long-shot risk probability *p*. However, the effect was entirely independent of population size. Over a 4-fold range of *n_max_* the survival probability curves were superimposable (not shown). The degree of risk aversion could be quantified by determining the amount that the native, intrinsic fecundity *g*_0_ of “R” needed to be increased in order to restore parity in the competition. This “risk premium” is plotted in Figure 3 (dashed line) as a function of the risk-reward *r*, for a fixed long-shot probability *p* = 0.1. Clearly, a gene for gambling would not be positively selected, in the absence of some other advantage. As discussed below, this unexpected, intrinsic risk aversion of stochastic selection stems from the fact that population growth factors *w* in successive generations are not statistically independent, because fluctuations in fecundity are negatively correlated with the population saturation (competition) factors in subsequent generations. As a result, the long-term expected growth rate differs from that in a single generation, to the disadvantage of the species with a higher variance in fecundity. Figure 2C shows the analytically-computed expected population growth after 1 and 2 generations, showing risk aversion that appears only in the second generation.

This situation was radically altered if the model was changed so that a small fraction of the reproductive reward or penalty earned by an individual organism from risk-taking could be passed directly to the next generation. This was implemented by starting each organism with a baseline fecundity (1−α) *g*_0_ + α *g_parent_* where *g_parent_* is the achieved fecundity, including risk reward or penalty (but before population-size scaling) of its parent. This kind of non-genetic inheritance of a parent's experience is actually entirely commonplace in the human population, in the form of inheritance of property and acquired knowledge. This culturally inherited wealth may increase the reproductive potential of offspring in the next generation. Any species with overlapping generations that directly supports or assists its own young might show a similar effect of parent's success on offspring's fecundity.

**Figure 4:**
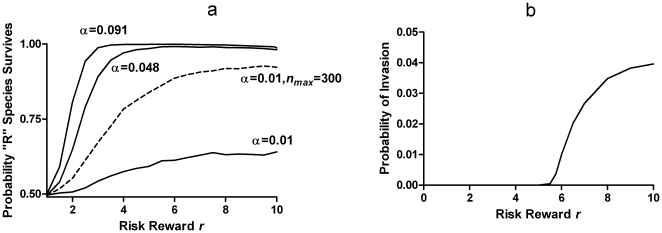
Selective advantage of risk-taking in the presence of “cultural inheritance”. a. Fraction of selection trials won by the “R” species, as in Figure 2A, but for the case where an “R” organism inherits a fraction α of the reproductive advantage or disadvantage obtained by its parent, before undergoing its own risk event. Note that there is a slight droop in the curves at the highest risk levels, indicating that the intrinsic risk aversion of due to population fluctuations is still active as in Fig. 2A,B, but is overcome by the accumulation of “inherited wealth” even at the smallest value of α The dashed line shows the effect of doubling the population size, which increases the selective pressure of “wealth” relative to drift. b. Probability that a single “R” organism with *g*_0_ = 1.5 will invade and replace a population of 149 “A” organisms with *g*_0_ = 2, despite the “R” having 25% lower expected reproductive fitness (α = 0.091, *p* = 0.1).

Figure 4 shows that, when this “cultural inheritance” was present, the “gambling” species was strongly favored. The “risk premium” became, instead, a discount (Figure 3, solid curve). In other words, even the propensity to take an unfair gamble would still be positively selected. At high levels of reward *r* the discount was so steep that the baseline fecundity of “R” could be reduced below replacement and the species would still survive and prevail, entirely on the basis of the reproductive advantage an organism acquires from a winning parent in the previous generation.

## Discussion

The fact that, in the absence of non-genetic inheritance, the risk-averse species is intrinsically favored is counterintuitive and has a somewhat subtle explanation. The single-generation growth factor *w* = *g_parent_*/(1+*n*/*n_max_*) is the ratio of two stochastic variables that are statistically independent, since the population at the start of a generation has no correlation with the outcome of the risk event. The expected single-generation growth is thus the same for both species, since the expected value of *g_parent_* is unaffected by risk (by construction) and the denominator is symmetrical in the two species. One might anticipate that the expected growth over multiple generations would be simply the product of the expected growth factors in every generation, but this is not correct. While *g_parent_* is independent of the population in its own generation, it is not independent of the population in the following generations, for obvious reasons. The growth factors in different generations are thus non-independent, so the expectation of the product of single-generation growth factors is not the same as the product of the expectations. In fact, because the population saturation function 1/(1+*n*/*n_max_*) is concave, the long-term expected growth is less, by an amount that increases with the variance of *g_parent_*, a fact which was confirmed by direct calculation for the 2-generation case (Figure 2C). Therefore, the lower-variance (*i.e.* risk-averse) species is systematically favored. It is at first surprising that this risk-aversion is independent of population size. One might expect this fluctuation-driven effect to decrease in larger populations, for which fluctuations in population size become smaller relative to the total. However, the risk effect acts as a bias of the random drift which is the mechanism of extinction in otherwise equivalent organisms. While the bias becomes smaller per generation, the number of generations required for drift to produce fixation of one organism is much larger in a large population, so that the bias has longer to act, leading to the same probability of the eventual outcome, as long as there is no systematic selective force.

The risk-advantage produced by “cultural inheritance” is due to a different kind of intergenerational correlation. The transmission of property or culture (intellectual property) induces a positive correlation between the reproductive fitness of parents and offspring. The expectation of the product of fitnesses over multiple generations is therefore *larger* than the product of the single-generation expected fitness, by an amount that increases with the size of fluctuations, favoring the species with the higher variance. Although the mean winnings of risk-takers is zero, or even negative, the next generation of “R” is disproportionately made up of children of winners, so they receive a net positive inheritance. To make the example concrete, imagine a polygamous society in which there is available a very risky financial opportunity with a 10% chance of multiplying the investment 10-fold, but a 90% chance of bankruptcy. Ten risk-averse men can each support one wife, and leave one son each. Ten men with a risk-taking gene on the Y-chromosome invest their life-savings; nine go bankrupt and cannot afford a wife and children, while one receives a windfall enabling him to support a harem that gives him 10 sons. In the second generation there are again 10 risk-averse men who will each have one son, and there are still 10 risk-taking men, each of whom has inherited one tenth of his father's winnings in addition to his own savings. One of these receives a windfall – now twice that of his father – enabling him to support a harem that gives him 20 sons. Thus, in the third generation, 2/3 of the population now carries the risk taking gene, and the process continues.

**Figure 5:**
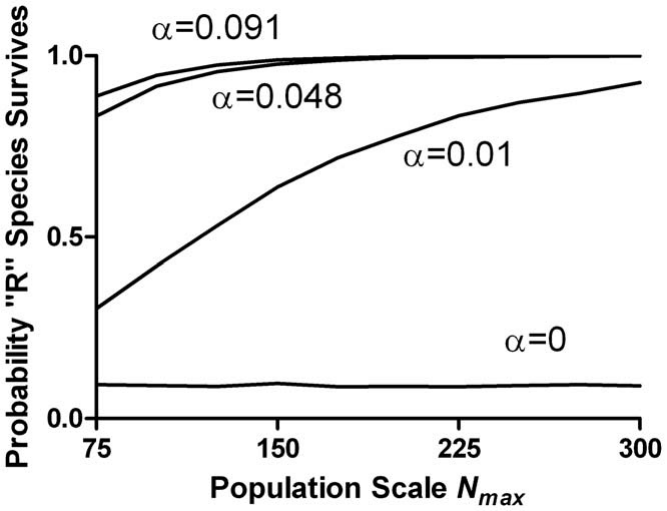
Effect of population size on selection for or against the risk-taking species. The fraction of trials in which the “R” species prevailed, at the maximum risk level (*r* = 10, *p* = 0.1) as a function of the population size parameter *n_max_* for several values of the “cultural inheritance” rate α, or in the absence of cultural inheritance (α = 0). When α = 0 there is no systematic effect of population size, because the “intrinsic risk aversion” due to the negative correlation of population-size fluctuations between generations scales in the same way as drift. When α>0 the positive inter-generational fecundity correlation competes with intrinsic risk aversion, prevailing in larger populations and higher values of α.

As shown in Figure 4a (dashed curve), this risk-advantage, unlike the intrinsic risk aversion in the purely genetic case above, is *not* independent of population size, but instead increases with population. This is because the correlation between fecundity fluctuations of parent and offspring, unlike that due to population fluctuations, does not decrease with population size, making it a systematic selective force. The effect of population size is shown in Figure 5 for several values of α, at the maximum risk level *r* = 1/*p*. The positive correlation effect due to cultural inheritance competes with the negative correlation effect due to population fluctuations (which scales like drift). The careful reader will also notice a very slight droop in the curves in Figure 4a at the highest risk levels due to this competition.

The effect of the positive correlation is cumulative over generations. This requires further comment in relation to the concept of fitness. The model has been constructed so that the expected number of offspring is either unaffected by taking the gamble, or – in the case of an unfair gamble – is actually reduced. In particular, the expected number of offspring of “R” is the same or less than that of “A” in the first generation. However, the operation of the cultural inheritance mechanism will result in the accumulation of “family wealth”, so that, in later generations, the expected number of offspring of “R” will be higher than that of “A”. It is misleading to think of this as implying that risk-taking increases individual fitness in the usual sense. For an “A” individual offered an unfair gamble, the rational strategy to maximize the expected number of his own offspring would be to save his inherited wealth in a risk-less asset and “mutate” to become risk-averse. However, if he did so, his family wealth would dissipate over a few generations and his line would become extinct in competition with continuing risk takers. The family wealth is maintained, not by any fitness advantage of gambling, but by the statistical correlation between gambling success and natural selection in the following generations.

This reveals an ambiguity in the concept of evolutionary fitness. The true fitness of an organism is the *a posteriori* realized number of offspring that it contributes to the next generation, in its actual environment (including the effect of other organisms). In this sense, evolution is indisputably the result of higher fitness operating over many generations. But a single organism does not evolve. When we speak of the fitness of an individual organism, there is implied some kind of *a priori* expectation value. As shown by our examples, when risk is involved, an expectation taken in a single generation does not successfully predict the outcome of selection, because it neglects inter-generational correlations, due either to population size fluctuations or to cultural or epigenetic effects. Therefore, we would favor Darwin's term “natural selection” over Spencer's “survival of the fittest.”

The accumulation of family wealth in the “R” population can be estimated. Starting with a population of “R” whose average fecundity in the *i*'th generation is <*g*_i_>, we estimate the number of winners and losers, and the respective numbers of offspring of winners and losers, and the values of *g* that they inherit, and then average those values over the next generation to obtain <*g*_i+1_>. After some algebra, the result is:
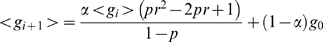



This is a linear recurrence relationship (finite-difference equation) for <*g*> describing the accumulation of average fecundity “wealth” over generations. As long as α<*p*, the coefficient of the linear term on the right will be less than 1 for all allowed risk levels (*r*<1/*p*) so <*g*> will converge asymptotically to a steady value higher than *g*_0_. If α>*p* there will be risk levels for which wealth increases exponentially over generations, which might make sense in financial terms but is unrealistic in terms of fecundity. To keep things simple and maintain explicit symmetry between the two species, the competition model was set up in such a way that the carrying capacity is proportional to the expected fecundity. As a result, in the presence of cultural inheritance, accumulated wealth can substantially increase the saturation population size. This is generally in keeping with the human experience, but since large increases in individual fecundity are unrealistic, we examined also a model in which the saturation denominator was computed from the sum of *g*−1 (at birth, before the risk event) rather than the physical number of organisms. This is tantamount to assuming that wealth places a proportional burden on resources. In this version, the saturation population stayed very close to *n_max_* but the results (not shown) were otherwise virtually identical to those shown above, the only difference being an increase in the slight “droop” in Figure 4 at the highest risk levels, due to a greater drift effect in the smaller population.

There is an intriguing, real-world case that bears a surprising degree of resemblance to our cartoon example of polygamous risk-takers. Zerjal *et al* [17] found a unique cluster of Y-chromosome haplotypes distributed throughout northern Asia at the extraordinary frequency of 8% coextensive with the boundaries of the former Mongol Empire founded by Genghis Khan. They provided evidence that this is, in fact, the Y-chromosome of Genghis Khan and his close male relatives, amplified enormously by “social selection” as a result of their founding of long-lived male dynasties whose rulers had very large numbers of children and passed their power (culturally) down the direct male line for centuries There is presently no evidence for a risk-taking gene on the Y-chromosome, but the early Mongols were notoriously daring. Since it is estimated that ∼4% of autosomes and 2.7% of X-chromosomes in the region are also descended from Genghis Khan, it might be fruitful to look for an excess of risk-taking genes in that population.

### Limitations

The results from our simplified model must be applied with caution to real biological and social situations, because of three kinds of limitations: (1) human and animal risk-taking is complicated and controlled by many genes; (2) there could be other evolutionary processes that would lead to apparently irrational risk sensitivity; (3) recombination in a sexually reproducing species might substantially affect the dynamics of selection.

1. Risk-sensitivity in man and other animals is complex, involving sequential life-history choices with genetic and environmental components and a strong interaction between the two [12]. In social species, selection for risk-taking behavior is also likely to be coupled to group interactions, hierarchy, altruism, etc. The evidence for irrationality in human risk sensitivity goes beyond gambling against the odds, and includes the “framing” effect, in which the willingness to take risks depends on whether they are presented in terms of gain or loss – an asymmetry that cannot be explained by any form of utility maximization [18]. Framing is sensitive to social and ethical value judgments (see [19] for a nice summary of the literature on this point). The risk-processing mechanisms in the brain responsible for such phenomena are a subject of active study. The kinds of heuristics used by the brain to respond to risky choices may be constrained as much by the limitations of neuroanatomy and physiology as by the evolutionary fitness effects of risk.
  While our model does not prove the *necessity* of non-genetic inheritance for the evolution of risk-seeking, the powerful effect it exerts in this simplified model suggests that it needs to be taken into consideration whenever cultural or epigenetic inheritance is present in more realistic situations. We modeled only symmetrical competition in which the reproduction of all individuals is equally depressed by the overall population size. Therefore, there is only one ecological niche, and no chance for the formation of a stable behavioral polymorphism of the kind that has been proposed as an explanation for animal personalities [20]. Our model differs also from the approach taken in evolutionary game theory [21]–[22] in which there is explicit competition between individuals in the same generation, so that fecundity gain by one organism represents a loss for others. In the extreme case of “winner take all” competition, such as the competition for mates among lekking birds, long-shot risk taking can be favored [23] because in a sufficiently large population there will always be at least one winner, who gets to father the entire next generation (this may be regarded as a convex nonlinear relationship between “earnings” and fecundity, analogous to the starving-animal paradigm in risk-sensitive foraging theory).
2. To know whether a gamble is a fair deal requires the organism to estimate quantitively the small probability of winning, and to effectively multiply this small number by the large payoff; neither of these quantitative operations is very practical in the natural world. An organism in an environment where most long-shot risks have positive expected payoff might evolve a simpler strategy: Always take the risk. Such an organism would perform disastrously when placed in a casino, but that would be merely a side effect of adaptation to a different environment. However, our model shows that even in an environment where every risk is a losing proposition in individual fitness terms, risk-taking can still be subject to positive selection if there is non-genetic inheritance.
3. Our simulation methods can be applied to the more realistic case in which risk-seeking and risk-averse alleles compete within a single sexually-reproducing species. To do this requires consideration of a great number of different cases. The fecundity phenotype needs to be specified for all possible matings of homozygote and heterozygote winners and losers. In the case of cultural or epigenetic inheritance, the manner in which “wealth” is transmitted to offspring as a function of gender (and perhaps birth order) needs to be specified, as well as the effects of assortive mating, which is the rule in human populations in relation to culture and material wealth. These studies will be important before the theory can be compared to empirical data. Because of the large numbers of scenarios that need to be simulated, we have elected not to consider the sexual case in this paper. We can point out, however, that as long as the inheritance system is such that acquired fitness exerts a positive effect on the fitness of offspring, the correlations that favor risk-taking genes will exist.

While we have been motivated mainly by the human case, in which the propensity of some individuals to take unfair, long-shot gambles is most striking, a similar effect might occur in any species with overlapping generations. The contribution of parental nurturing to survival of the young is already taken into account in the definition of reproductive fitness of the parent (we conceptualize the life cycle as going from one reproductively competent adult to the next). However, any direct contribution of parental resources to reproductive success of the offspring in the next generation would have an effect similar to cultural inheritance in our model. Even in species without overlapping generations, parental effects transmitted through the egg can propagate parental experience to affect offspring fecundity [24]. Another form of non-genetic inheritance is the epigenetic modification of genes in the zygotes. It has been shown that environmental and nutritional influences can alter trans-generational epigenetic gene regulation [25]–[26]. This might potentially produce effects analogous to cultural inheritance. It is reasonable, then, to consider a role for non-genetic inheritance when interpreting risk-sensitivity of decision-making behavior across the animal kingdom.

## Materials and Methods

The stochastic selection algorithm was implemented in Fortran. The population of a species was represented by an array listing the initial fecundity *g* of each of the organisms. At the start this was set to *g*_0_ for all organisms, where this constant could be chosen separately for each species. One generation of reproduction consisted of the following: For each “R” organism, a uniform random number between 0 and 1 was chosen, and if it was less than a fixed probability *p*, the initial fecundity was multiplied by *r*, the risk reward factor (>1); otherwise it was multiplied by (1−*rp*)/(1−*p*) which is less than 1. This gave the organism's unconstrained fecundity *g_parent_* (*i.e.* the expected number of offspring that it would contribute to the next reproductive generation if there were not competition for resources). For the “A” species, the risk event was omitted. The actual, realized number *k* of offspring contributed to the next generation by an organism was chosen from a Poisson distribution with mean *w* = *g_parent_*/(1+*n*/*n_max_*) where *n* is the total number of organisms of both species present in the current generation. The organism was then replaced in the array by *k* entries “born” with initial fecundity (1−α) *g*_0_ + α *g_parent_*. For the case of purely genetic inheritance, α = 0. For the version of the model with constant carrying capacity, the total population *n* in the saturation denominator was replaced by the sum of (1−α) *g*_0_ + α *g_parent_*−1 over all organisms.

Starting from a population containing equal numbers of each species, the above generation cycle was repeated until the number of organisms of one species reached zero (extinction) as shown in Figure 1. This selection process was repeated 10,000 times to estimate the frequency with which “R” was the winning species, plotted in Figures 2A,B, 4a and 5. The probability of invasion (Figure 4b) was calculated similarly, but starting with *n_max_*−1 “A” organisms and only a single “R”. To calculate the risk premium, the *g*_0_ value of “R” was adjusted up or down manually until exactly 50% of the selection runs resulted in survival of “R”. The required change in *g*_0_ was plotted as a percentage in Figure 3.

The methods used in these simulations are analogous to the techniques used to predict the survival of endangered species in population viability analysis (cf. [27]). We have the advantage of being able to invent the demographic parameters instead of having to measure them over decades, and we have omitted such effects as age/stage stratification, spatial variability, sub-populations, migration and continuous-time demographic events that will eventually need to be considered to extend our arguments to realistic species.

In order to demonstrate that the “intrinsic risk aversion of selection” arose from correlations between growth rate fluctuations in different generations, we computed analytically the expected population sizes after *i*_gen_ generations. This proved surprisingly difficult. It requires summing over all the possible population paths defined by the numbers of offspring of each organism in each generation, weighted by their probabilities, an impossible task. Fortunately, our model represented risk as a simple dichotomy (win *vs.* lose) which enables the state of the population to be described by a single binomial distribution, and the stochasticity of individual reproduction was represented by a Poisson distribution, which has the convenient property that the sum of multiple Poisson variates is again Poisson. This allowed lumping of organisms into three pools – winners, losers and risk-averse, by which means we arrived at the following recursive equation for the expected number of “R” individuals after *i*_gen_ generations, starting with *n_r_* risk-taking and *n_a_* risk-averse organisms in the first generation:
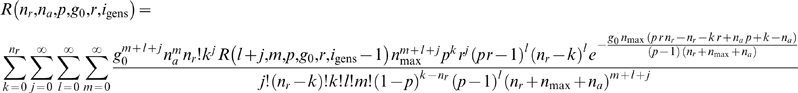
with the understanding that when *i*_gen_ = 0, *R* = *n_r_*. A similar function applies to “A”. The operation of this equation may require some clarification. At entry to the “top level” of function *R* on the left, the function is presented with the integer numbers of “R” and “A” organisms in the first generation. The function considers all possible integer numbers of winners and losers and the possible integer numbers of of offspring they might produce at the end of the first generation. The probabilities of all these possible second-generation populations are computed and used as weighting factors multiplying the (non-integer) expected numbers of organisms after the remaining *i_gen_*−1 generations. The latter is computed by a second, recursive call to the function *R* itself. The top level of this call receives as input the integer numbers of “R” and “A” in (each of) the hypothetical second generations. The number of remaining generations is decremented by one on each successive nested call, until, when there are no generations left, the innermost call of *R* simply returns the population size it was presented with. At each level, an independent quadruple sum is carried out over the possible productions of that generation, so that eventually the top level of *R* returns an average of the final population size taken over all possible intermediate population histories weighted by their likelihood. In each probability computation, the saturation denominator is calculated using the integer number of organisms present in that hypothetical generation, so that the fluctuations in competition are fully accounted for.

This function was computed numerically by a recursive Fortran subroutine. The comparatively compact form of the equation belies the huge number of individual function evaluations, which increases exponentially with the number of generations, and approximately as the 8^th^ power of the population size when *i*_gen_ = 2. This limited numerical evaluation to one or two generations and a small population (10 of each species with *n_max_* = 20), which still required several hours of computer time on a 3 GHz Pentium. This proved sufficient to demonstrate the principle (Figure 2C). Even though the expected output of the second generation was the same for both species for each possible input coming from the first generation, the two-generation expected growth rate differed, due to correlation between the fluctuations of realized *w* between generations.
